# Artichoke phytocomplex modulates serum microRNAs in patients exposed to asbestos: a first step of a phase II clinical trial

**DOI:** 10.1186/s13046-022-02455-6

**Published:** 2022-08-20

**Authors:** Paola Muti, Andrea Sacconi, Claudio Pulito, Giulia Orlandi, Sara Donzelli, Aldo Morrone, James Jiulian, Gerard P. Cox, Martin Kolb, Gregory Pond, Peter Kavsak, Mark Norman Levine, Giovanni Blandino, Sabrina Strano

**Affiliations:** 1 Department of Biomedical, Surgical and Dental Sciences, University of Milan, Milan, Italy; 2 Department of Oncology, McMaster University, Hamilton, ON Canada; 3 Biostatistics and Bioinformatics, UOSD Clinical Trial Center, IRCCS Regina Elena National Cancer Institute, Rome, Italy; 4 Translational Oncology Research Unit, Department of Research, Advanced Diagnostic, and Technological Innovation, IRCCS, Regina Elena National Cancer Institute, Rome, Italy; 5 Scientific Direction Office, IRCSS San Gallicano Dermatological Institute, Rome, Italy; 6 Juravinski Cancer Center, Hamilton Health Science, Hamilton, ON Canada; 7 Ontario Clinical Oncology Group, Hamilton, ON Canada; 8 Department of Medicine, McMaster University, Hamilton, ON Canada; 9 Department of Pathology and Molecular Medicine, McMaster University, Hamilton, ON Canada; 10 SAFU Laboratory, Department of Research, Advanced Diagnostic, and Technological Innovation, IRCCS Regina Elena National Cancer Institute, Rome, Italy

**Keywords:** Asbestosis, Malignant pleura mesothelioma, Artichoke, Biomarker, miRNA, Mesothelin

## Abstract

**Background:**

Malignant pleural mesothelioma is a highly aggressive tumor associated with asbestos exposure. There are few effective treatment options for mesothelioma, and patients have a very poor prognosis. Mesothelioma has the potential to represent an appropriate disease to prevent because of its strong association with asbestos exposure and the long latency from exposure to the disease on-set.

**Methods:**

In the present study, we tested biological activity and toxicity of an artichoke freeze-dried extract (AWPC) as potential complementary preventive/early stage treatment agent for mesothelioma. This phase II clinical study then was conducted in 18 male-patients with evidence of radiographic characteristics related to asbestos exposure such as asbestosis or benign pleural disease as surrogate disease for mesothelioma clinical model.

**Results:**

We investigate AWPC biological activity assessing its effect on mesothelin serum level, a glycoprotein with low expression in normal mesothelial cells and high expression in mesothelioma and asbestos related diseases. We also assess the AWPC effect on circulating miRNAs, as novel biomarkers of both cancer risk and response to therapeutic targets. While we found a small and not significant effect of AWPC on mesothelin serum levels, we observed that AWPC intake modulated 11 serum miRNAs related to gene-pathways connected to mesothelioma etiology and development. In terms of toxicity, we also did not observe any severe adverse effects associated to AWPC treatment, only gastro-intestinal symptoms were reported by five study participants.

**Conclusions:**

We observed an interesting AWPC effect on miRNAs which targets modulate mesothelioma development. New and much larger clinical studies based on follow-up of workers exposed to asbestos are needed to corroborate the role of AWPC in prevention and early treatment of mesothelioma.

**Trial registration:**

ClinicalTrials.gov, NCT02076672. Registered 03/03/2014.

**Supplementary Information:**

The online version contains supplementary material available at 10.1186/s13046-022-02455-6.

## Background

Malignant Pleural mesothelioma (MPM) is an asbestos-related cancer characterized by a five-year survival rate of approximately 12% [1]. The predominant cause of malignant mesothelioma is asbestos inhalation, with approximately 70% of cases associated with documented asbestos exposure. Inflammation, free radical production and direct DNA damage are recognized pathogenic features of asbestos exposure [2]. Asbestosis and pleural plaques are non-malignant pleural diseases that result from fibers reaching both lung and pleura sharing the same asbestos etiological risk factor [3].

Currently nothing is being done to lower individual cancer risk due to asbestos exposure. Chemoprevention is a potential avenue for risk reduction in asbestos exposed individuals. However, up to now, very few trials have been undertaken and reported. The global demand for affordable anti-cancer agents has renewed interest in the use of naturally occurring molecules with chemopreventive and chemotherapeutic properties for cancer prevention programs.

Artichoke leaf, a medicinal plant known for its choleretic, antioxidant, anti-dyspeptic and anti- dyslipidemic activities, has been investigated by our group for activity against activation of STAT3 nuclear transcriptional factor, involved in the initiation and progression of mesothelioma. We found that artichoke leaf freeze-dried extract had activity in decreasing viability, invasiveness and chemo-resistance of malignant mesothelioma cells [4].

We tested Artichoke freeze-dried extract (Abo-1) in a single-arm cohort study (OCOG-2013-ABOCA, NCT number NCT02076672) aimed at assessing the biological activity and toxicity of this supplement in patients with evidence of radiographic characteristics related to asbestos exposure and consistent with a diagnosis of non-malignant asbestos-related disease, either asbestosis or benign pleural disease. The choice to include patients with benign asbestos related lesions, at increased risk of mesothelioma, instead of MPM was due to the extreme severity of mesothelioma cases admitted to the surgical ward which prevented us to implement a compliant research study.

The primary efficacy outcome was based on the reduction in serum concentration levels of mesothelin, measured at baseline and after a 90-day treatment. Serum mesothelin is a protein produced by mesothelium in response to acute and chronic states of inflammation and associated with mesothelioma development [5]. The secondary outcome of the study was to assess whether circulating miRNAs were also related to treatment response. miRNAs exhibit tumor-specific expression profiles and have been observed in both cancer patients’ and healthy controls’ serum. Several studies have addressed the potential association of dysregulated miRNA profiling and benign asbestos-related disease and mesothelioma [6, 7]. Finally, the safety outcome was the detection of adverse reactions (AR) due to the drug during the reporting period that began at the time of the administration of the first dose of study medication and ended 30 days after the last dose of study medication.

## Methods

### Design

The study was a single-arm cohort study designed as a Simon two-stage Phase II design in patients with either asbestosis or benign pleural disease with mesothelin serum level higher than 0.40 nmol/L. The study intervention was AWPC. The primary outcome measure was reduction is serum mesothelin. The secondary aim was miRNA modulation. The study was coordinated by the Ontario Clinical Oncology Group. (NCT number NCT02076672).

### Patient population

#### Recruitment

Participants were recruited from the practices of pulmonary physicians and thoracic radiology at the Firestone Clinic at St. Joseph’s Hospital in Hamilton, Ontario under the supervision of Dr. G. Cox and Dr. M. Kolb. After confirmation of eligibility and documentation of written informed consent, patients were registered and enrolled into the study, by the clinical center, using the web-based IRIS system maintained by the OCOG Coordinating and Methods Centre located at the Juravinski Hospital in Hamilton, Ontario, Canada.

The inclusion criteria were as it follows: a) having evidence of radiographic characteristics related to asbestos exposure and consistent with a diagnosis of non-malignant asbestos-related disease, either asbestosis or benign pleural disease; b) having mesothelin serum level higher than 0.40 nmol/L.

The main exclusion criteria were any prior cancer, any biliary condition for which AWPC was a contraindication, and mental disorders.

The study was approved by the Hamilton Integrated Research Ethics Board and all participants signed a written informed consent. The study product, AWPC, was approved by Health Canada (Reg. Num. HC6-24-c170190).

The baseline assessment included the collection of patient demographics, exposure to asbestos (yes/no, estimate of years of exposure), cigarette smoking (pack-years), medical history and documentation of concurrent medications. Physical exam included measurement of height and weight, heart rate and blood pressure. Routine blood work (i.e., CBC, platelet count, creatinine, bilirubin, ALT or AST) was also collected.

### Study intervention

#### Treatment

AWPC is derived from artichokes (Cynara Scolymus) for which the main components are caffeoylquinic acid derivatives (cynarine and chlorogenic acid), flavonoids (luteolin and apigenin) and bitters (cynaropicrin). The study agent is based on the commercially available AWPC capsules. This product contains 30% of the artichoke freeze-dried extract, used in all the preclinical experiments [4], and 70% of micronized powder from the same portion of the artichoke plant (Cynara scolymus L.) leaves. The micronized powder is used to stabilize the active freeze-dried extract and to protect it against degradation. As described in the preclinical documentation, the highest artichoke extract dose successfully used to treat mice was 100 mg/kg. Following the FDA conversion table (ref: www.fda.gov), this concentration converts to an 8.13 mg/kg human-equivalent dose. Assuming a median body weight of 90 kg in the target population, we calculated an artichoke extract dose of 732 mg per day. Each 500 mg capsule of AWPC contains 404 mg of product (121 mg of artichoke extract and 283 mg of micronized artichoke leaf powder). To achieve this dose of extract extrapolated from the preclinical data, we needed to administer 6 capsules (i.e., 3000 mg) per day of AWPC to each patient. Study subjects remained on AWPC therapy for 90 days and were monitored for toxicity on Day 45 and at the end of the study Day 90. Treatment compliance was assessed by pill count. Adherence was defined at each study visit as the use of at least 85% of pills.

### Assays

#### Blood samples

Blood was collected at baseline and at the end of the treatment period (Day 90) for serum mesothelin levels and miRNAs assessment. After drawing, blood was centrifuged and then separated in 6 different 1.7 ml samples and stored at -80 °C until mesothelin and miRNA assessment. Mesothelin (nmol/L) was measured in serum using the MESOMARKTM assay (Cis Bio International, Gif sur Yvette, France). The estimated coefficient of variation (CV) at the levels observed in this study was 2.5% for the method detection level is 0.30 nmol/L. Based on the method detection level of 0.30 nmol/L, to detect > 25% reduction in baseline mesothelin, as absolute change between 90th day of treatment and baselines values, we included subjects with levels > 0.40 nmol/L. Serum Mesothelin was assessed in different batches and in blind condition in respect of the baseline serum concentration.

For miRNA determination, matched samples (baseline and end-of-the study) from the same individual were retrieved, thawed, and assessed together and by the same laboratory technician to control, at least in part, for effect of technical variability.

#### Plasma RNA extraction

Human plasma specimens were processed within 2 h from blood collection, centrifuge at 3000 rpm for 15 min and stored at − 80 °C. Total RNA from plasma specimens was extracted by the miRNeasy Serum/Plasma Kit (Qiagen, Valencia, CA, USA) according to the manufacturer’s instructions.

MiRNAs Hybridization. 200 ng of total RNA for each sample was used to generate fluorescent microRNA by using the Agilent’s microRNA Complete Labeling and Hyb Kit (Agilent) according to manufacturer’s instructions. Labeled RNA was hybridized to human microRNA Microarray V21 (Agilent), containing probes for 2570 human miRNAs. Scanning and image analysis were performed using the Agilent DNA Microarray Scanner (P/N G2565BA). Feature Extraction Software (V-10.5) was used for data extraction from raw microarray image files using the microRNA_105_Dec08 FE protocol.

### Study outcomes

The primary outcome was the decrease in mesothelin serum levels after treatment with AWPC for 90 days. The secondary outcome was the assessment of circulating miRNA expression modulation between the baseline and after the 90 day-period of treatment.

Toxicity was assessed using the NCI CTCAE version 4.03. For each reaction, the highest severity grade attained since the last assessment period was reported. If a CTCAE category did not exist, the Investigator assessed the AR as Grade 1 (mild), Grade 2 (moderate), Grade 3 (severe), Grade 4 (life-threatening), Grade 5 (death) to describe maximum intensity of the adverse reaction.

### Statistics

#### Sample size

The sample size calculation was based on the estimated proportion of subjects who had a baseline mesothelin level > 0.40 nmol/L, and Simon’s optimal two-stage Phase II design. This is a single-arm study that tests the null hypothesis of insufficient efficacy versus an alternative that the treatment has sufficient activity to merit further investigation. In our study, the treatment effect on mesothelin has been estimated using the results of Creaney et al., 2011 where a positive outcome would be a decrease of mesothelin serum levels by 25% or more after treatment [8]. If the proportion of patients with a positive outcome was expected to be less than or equal to 15%, the treatment was considered not sufficiently promising, and the trial would have been stopped. On the other hand, a response of 35% or more would be a desirable level for pursuing this product in a later phase trial. We have set α = 0.10 and β = 0.10, thus the optimal sample size for the first step of the study was 19 patients. Starting from this sample size, if only 3 or fewer patients would have responded, the treatment did not prove a substantial biological activity with the consequent interruption of the trial at this first stage. If the treatment would have created a positive response in 4 or more patients, then some biological activity was evident with a consequent continuation to the second stage with the additional recruitment of 14 patients. At that point, if only 7 or fewer patients would have responded out of the total of 33 (< 24%), the treatment would have been considered to provide too low biological activity to justify an extension to a phase III trial.

Based on mesothelin first step results, we decided to continue the trial to the second stage. However, the active patient recruitment period for the second stage brought us to the beginning of the Sars-Cov-2 pandemia with the enrollment closure at the Firestone Institute for Respiratory Health. Thus, the present report includes only all 18 patients participating in the first step of the study.

In order to validate miRNA results from the trial, we included a cohort of 41 patients affected with either mesothelial benign lesions or mesothelioma which data were previously published by our group [9]. In that study, we performed an unbiased microRNA screening of malignant pleural mesothelioma specimens (*n* = 29) and mesothelial benign tissues (mesothelial cysts, *n* = 12). The samples were collected during surgery from a series of patients admitted at the Italian National Cancer Institute (INCI) between 2009 and 2012.

#### Analysis

Descriptive statistics including 95% confidence intervals were used to summarize study variables. For continuous measures, we used means, standard deviations, quartiles, minimum and maximums. For variables with skew distributions, we employed a log transformation prior to summarizing. For categorical variables, we used counts and percentages as summary measures. Relationships between continuous measures (e.g., baseline and post-treatment mesothelin values) were explored with scatterplots; for categorical outcomes, we used cross-tabulations. Statistical modelling of the change scores were performed using linear models with adjustment for degree of asbestos exposure, and other baseline factors such as the severity of radiological signs of benign asbestos-related disease, cigarette smoking and age.

#### Microarray data analysis

Data were verified and extracted by the Agilent Extraction 10.7.3.1 software and analyzed using an inhouse built routines by Matlab R2020b. (The MathWorks Inc.). Background-subtracted signal of 2571 human miRNA assays was used in the study. All arrays were quantile normalized, assuming that all samples were measured and analyzed under the same condition, enforcing all the arrays to assume the same mean distribution.

MiRNAs expression for 18 no-treated (baseline) and 18 treated (end-of-the study) matched samples from Agilent platform were analyzed. Significantly modulated miRNAs were assessed by using a permutation test and a paired-wilcoxon test. ROC analysis was performed and miRNAs with AUC (Area Under Curve) less than 70% were excluded from further evaluations.

#### In silico analysis

The MiRNet web tool (https://www.mirnet.ca/miRNet/home.xhtml (accessed on 12 May 2022)), based on Tarbase v8, was used to determinate miRNA-target interaction. The graphical view of the network built by the validated targets of the 11 differently expressed miRNAs signature or miR-181a-5p and miR-193a-5p was obtained by using Cytoscape software. The enrichKEGG R function of clusterProfiler package was used to identify and graph the pathways analysis. HALLMARK pathways from MsigDB obtained using the web tool ShinyGO. The enrichment has been separately evaluated by considering predicted targets of the 11-miRNA signature and the putative targets of the first 15 miRNA modulated between malignant mesothelioma and benign cysts (IRE cohort).

## Results

### Mesothelin

For Stage 1 of the trial, 22 patients, all males, were recruited between November 2014 and July 2015. All patients satisfied the eligibility criteria. Of these 22 participants, 4 withdrew: one did not return after his baseline visit (#5), and 3 withdrew during the 90 days due to ARs (#18, #19, #20). Thus, the study was conducted on a total of 18 patients. The baseline characteristics of the 22 subjects are shown in Table 1. Most of the study patients were affected by asbestos related benign plaques and all of them have been exposed to asbestos fibers for a median exposure time of 10 years.

**Table 1:** Patients baseline characteristics

Characteristic	Completed Trial (*n* = 18)	Withdrew (*n* = 4)
**Age**, *yrs: median, range*	72 (64–79)	67 (63–72)
**Height**, *cm: median, range*	172 (164–180)	173 (168–180)
**Weight**, *kg: median, range*	81 (68–108)	86 (78–108)
**BMI**, *kg/m*^*2*^*: median, range*	26.7 (21.0–37.5)	29.0 (26.7–33.3)
**Disease Type**: *n (%)*		
Asbestosis	2 (11)	0
Pleural Plaques	16 (89)	4 (100)
**Industry Type**: *n (%)*		
Factory	6 (33)	3 (75)
Mechanical	6 (33)	0
Construction	3 (17)	1 (25)
Shipbuilding	2 (11)	0
Office	1 (5)	0
**Asbestos Exposure**, *yrs:* *median, range*	10 (1–48)	10 (1–27)
**Smoking**: *n (%)*		
Never	4 (22)	0
Quit (> 6 months ago)	12 (67)	3 (75)
Current	2 (11)	1 (25)

Patient-specific levels of mesothelin (in nmol/L) at baseline, and at 90 days, for the 18 analyzable subjects are shown in Table 2. To keep costs down, the baseline blood samples of the 4 who withdrew were not sent for analysis. Seventeen of the 18 patients had baseline levels above 0.40 nmol/L; only one patient had a mesothelin level of exactly 0.40. Because the first stage of the trial required 19 patients, and since we had only 18 analyzable subjects, we decided to include the one patient with a 0.4 baseline level. Overall, 5 subjects showed a decrease in mesothelin levels (28%), 7 showed no change (39%) and 6 showed an increase (33%). Of the 5 with lower levels at Day 90, only 3 met the definition of a study success (i.e., decrease of 25% or more). A 4th patient had the largest absolute decrease of 0.6 nmol/L (from 2.6 to 2.0). Although this was a decrease of only 23.1% in relative terms, we felt compelled to call this a success as well (4/18 = 22%). Based on these results, we decided to continue for the second stage of the study which was halted because of COVID-19 pandemic.

**Table 2:** Mesothelin Results (nmol/L) at Baseline, Day 90 and the Change

	**#**	**Baseline**	**Day 90**	**Change**	**Change%**	**Direction**
	**6**	0.6	0.4	-0.2	**-33.3**	**Decrease**
	**1**	1.3	0.9	-0.4	**-30.8**	
	**10**	0.7	0.5	-0.2	**-28.6**	
	**11**	2.6	2.0	-0.6	**-23.1**	
	**12**	1.3	1.1	-0.2	**-15.4**	
	**4**	0.9	0.9	0.0	0	No Change
	**7**	1.2	1.2	0.0	0	
	**9**	0.7	0.7	0.0	0	
	**13**	0.5	0.5	0.0	0	
	**14**	1.2	1.2	0.0	0	
	**15**	1.1	1.1	0.0	0	
	**16**	1.7	1.7	0.0	0	
	**3**	0.8	0.9	0.1	12.5	**Increase**
	**17**	0.7	0.9	0.2	28.6	
	**21**	0.6	0.9	0.3	50.0	
	**2**	1.0	2.0	1.0	100.0	
	**8**	0.6	1.3	0.7	116.7	
	**22**	0.4	0.9	0.5	125.0	
**Minimum**		0.4	0.4	-0.6	-33.3	
**Median**		0.85	0.9	0.0	0.0	
**Maximum**		2.6	2.0	1.0	125.0	
**Mean**		0.99	1.06	0.07	16.8	
**Std. Dev**		0.53	0.46	0.38	49.3	

### MiRNA modulation

**Fig. 1:**
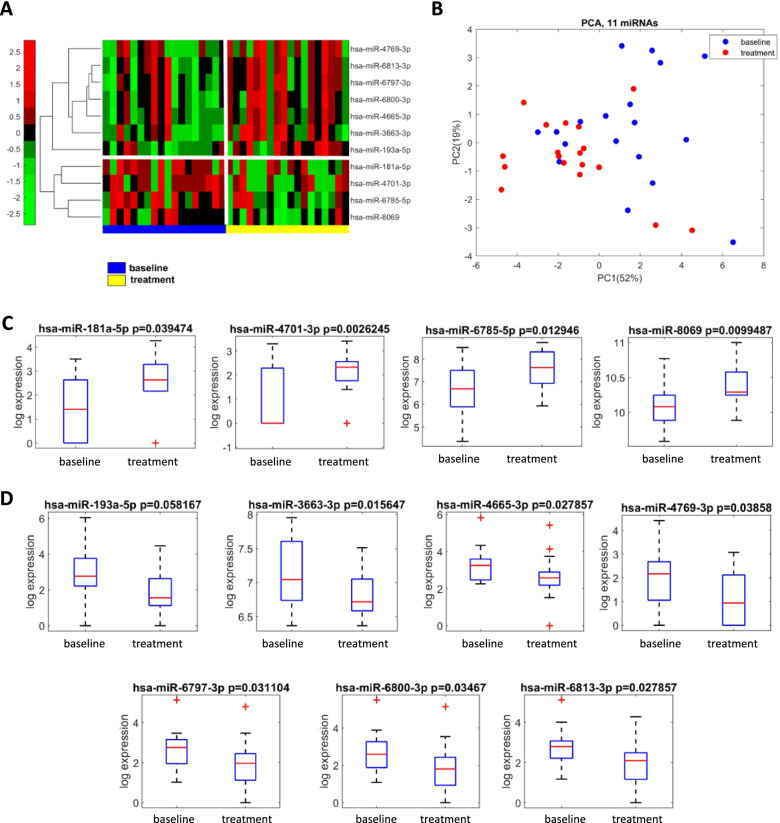
**A** Supervised hierarchical clustering representing differentially expressed miRNAs before (baseline) and after (treatment) artichoke leaf extract treatment. Red and green colours show high and low standardized expression levels, respectively. **B** Principal component analysis representation of samples before (baseline) and after (treatment) artichoke leaf extract treatment obtained from the 11 miRNAs signature. **C-D** Boxplot analysis representing the distributions of the 11 miRNAs signature. Differences in the miRNA expression are evaluated by Wilcoxon test

Figure 1A displays the heatmap of the 11 miRNAs (out of 2571 tested by the array platform) significantly dysregulated between the baseline and after the three months of AWPC treatment. The heatmap is separated by a vertical white line to make an easy visual comparison of the miRNA expression between the baseline and the end of the study. The horizontal line separates, on the contrary, those miRNAs down-regulated versus those up-regulated at baseline and at the end of treatment. Figure 1B displays the results of an unsupervised factor analysis using principal component solution based on the whole set of data under unsupervised strategy. The analysis confirmed the ability of the 11-miRNA signature to discriminate between the baseline data versus the post-treatment data. Plotting the data by PC1 and PC2, the post-treatment group of observation appeared to be more closely assembled then the baseline group suggesting a reduction of intra-group variability as an effect of AWPC treatment.

In Fig. 1C-D and Supplementary Table 1, we have reported the detailed figure of the significantly dysregulated miRNA expression and related *p* values between baseline and end of the study for those up-regulated (panel C for miR-181a-5p; miR-4701-3p; miR-6785-5p; miR-8069) and those down-regulated (panel D for miR-193a-5p; miR-3663-3p; miR-4665-3p; miR-4769-3p; miR-6797-3p; miR-6800-3p; miR-6813-3p).

Then, to understand the targeted functional characteristics of the identified 11 miRNAs, we proceeded in the analysis by looking at the Molecular Signatures Database (MSigDB). In Table 3 and Supplementary Fig. 1A, we have listed all the genes and related pathways associated with the considered miRNAs. Most of them were connected to cancer development, metabolism and inflammation: all are important pathways activated in mesothelioma development and progression [10].

**Table 3:** HALLMARK pathways from MsigDB obtained using the web tool ShinyGO. The enrichment has been separately evaluated by considering predicted targets of the 11-miRNA signature (List AWPC) and of the 15-miRNA signature (List Derived from Comparison Benign vs Mesothelioma lesions)

*List AWPC*			
**Functional Category**	**Enrichment FDR**	**Genes in list**	**Total genes**
MSigDB:HALLMARK ESTROGEN RESPONSE EARLY	8.00E-10	168	197
MSigDB:HALLMARK MTORC1 SIGNALING	8.00E-10	168	197
MSigDB:HALLMARK IL2 STAT5 SIGNALING	3.00E-09	165	195
MSigDB:HALLMARK INFLAMMATORY RESPONSE	8.17E-08	163	197
MSigDB:HALLMARK UV RESPONSE DN	8.17E-08	121	141
MSigDB:HALLMARK APOPTOSIS	1.14E-07	134	159
MSigDB:HALLMARK APICAL JUNCTION	1.14E-07	160	194
MSigDB:HALLMARK ANDROGEN RESPONSE	2.77E-07	86	97
MSigDB:HALLMARK PI3K AKT MTOR SIGNALING	4.38E-07	91	104
MSigDB:HALLMARK P53 PATHWAY	7.97E-07	159	196
MSigDB:HALLMARK HYPOXIA	1.07E-06	157	194
MSigDB:HALLMARK PROTEIN SECRETION	1.07E-06	84	96
MSigDB:HALLMARK MITOTIC SPINDLE	2.00E-06	159	198
MSigDB:HALLMARK GLYCOLYSIS	2.00E-06	159	198
MSigDB:HALLMARK IL6 JAK STAT3 SIGNALING	3.65E-06	76	87
MSigDB:HALLMARK TNFA SIGNALING VIA NFKB	8.82E-06	157	198
MSigDB:HALLMARK KRAS SIGNALING UP	9.12E-06	154	194
MSigDB:HALLMARK UV RESPONSE UP	2.07E-05	124	154
MSigDB:HALLMARK E2F TARGETS	9.99E-05	152	196
MSigDB:HALLMARK DNA REPAIR	1.58E-04	112	141
MSigDB:HALLMARK HEME METABOLISM	2.76E-04	151	197
MSigDB:HALLMARK G2M CHECKPOINT	3.81E-04	149	195
MSigDB:HALLMARK EPITHELIAL MESENCHYMAL TRANSITION	8.43E-04	149	197
MSigDB:HALLMARK TGF BETA SIGNALING	1.17E-03	46	54
MSigDB:HALLMARK KRAS SIGNALING DN	1.23E-03	144	191
MSigDB:HALLMARK MYOGENESIS	1.62E-03	149	199
MSigDB:HALLMARK COMPLEMENT	1.76E-03	146	195
MSigDB:HALLMARK ESTROGEN RESPONSE LATE	1.77E-03	148	198
MSigDB:HALLMARK UNFOLDED PROTEIN RESPONSE	2.38E-03	85	109
MSigDB:HALLMARK CHOLESTEROL HOMEOSTASIS	2.60E-03	59	73
*List Derived from Comparison Benign vs Mesothelioma lesions*			
**Functional Category**	**Enrichment FDR**	**Genes in list**	**Total genes**
MSigDB:HALLMARK UV RESPONSE DN	5.89E-11	128	141
MSigDB:HALLMARK IL2 STAT5 SIGNALING	1.54E-10	169	195
MSigDB:HALLMARK KRAS SIGNALING UP	4.73E-10	167	194
MSigDB:HALLMARK ESTROGEN RESPONSE EARLY	1.61E-09	168	197
MSigDB:HALLMARK MTORC1 SIGNALING	3.40E-08	165	197
MSigDB:HALLMARK ADIPOGENESIS	6.19E-08	159	190
MSigDB:HALLMARK PI3K AKT MTOR SIGNALING	3.23E-07	92	104
MSigDB:HALLMARK APICAL JUNCTION	3.23E-07	160	194
MSigDB:HALLMARK INFLAMMATORY RESPONSE	3.23E-07	162	197
MSigDB:HALLMARK HEME METABOLISM	3.23E-07	162	197
MSigDB:HALLMARK ANDROGEN RESPONSE	4.47E-07	86	97
MSigDB:HALLMARK PROTEIN SECRETION	5.62E-07	85	96
MSigDB:HALLMARK APOPTOSIS	1.38E-06	132	159
MSigDB:HALLMARK P53 PATHWAY	1.74E-06	159	196
MSigDB:HALLMARK TNFA SIGNALING VIA NFKB	2.41E-06	160	198
MSigDB:HALLMARK COMPLEMENT	4.42E-06	157	195
MSigDB:HALLMARK HYPOXIA	5.18E-06	156	194
MSigDB:HALLMARK EPITHELIAL MESENCHYMAL TRANSITION	5.69E-06	158	197
MSigDB:HALLMARK MYOGENESIS	1.66E-05	158	199
MSigDB:HALLMARK IL6 JAK STAT3 SIGNALING	1.82E-05	75	87
MSigDB:HALLMARK UV RESPONSE UP	1.82E-05	125	154
MSigDB:HALLMARK GLYCOLYSIS	1.39E-04	154	198
MSigDB:HALLMARK KRAS SIGNALING DN	1.39E-04	149	191
MSigDB:HALLMARK MITOTIC SPINDLE	2.53E-04	153	198
MSigDB:HALLMARK ESTROGEN RESPONSE LATE	4.51E-04	152	198
MSigDB:HALLMARK OXIDATIVE PHOSPHORYLATION	5.38E-04	141	183
MSigDB:HALLMARK PEROXISOME	7.41E-04	83	103
MSigDB:HALLMARK APICAL SURFACE	9.05E-04	38	43
MSigDB:HALLMARK G2M CHECKPOINT	1.16E-03	148	195
MSigDB:HALLMARK DNA REPAIR	1.78E-03	109	141

The results highlighted so far changes in the expression of miRNAs before and after treatment with AWPC in patients with either benign lesions induced by asbestos exposure or patients with asbestosis. To understand whether all or any of the 11 miRNAs modified by AWPC treatment were also potential targets for mesothelioma, we conducted a validation study. For the validation study, we first considered data from a different previously conducted study by our group [9]. In that study we assessed differences in miRNAs expression profiles between unmatched tissues from benign pleural lesions (same type we used as an inclusion criterion in the trial) derived from 12 subjects and mesothelioma-derived tissues collected from 29 patients. Our analysis revealed striking differences in the microRNA expression profile between benign vs malignant mesothelial tissues allowing us to identify 15 microRNAs which showed statistically significant dysregulation in benign vs malignant tissue (Supplementary Table 2).

**Fig. 2:**
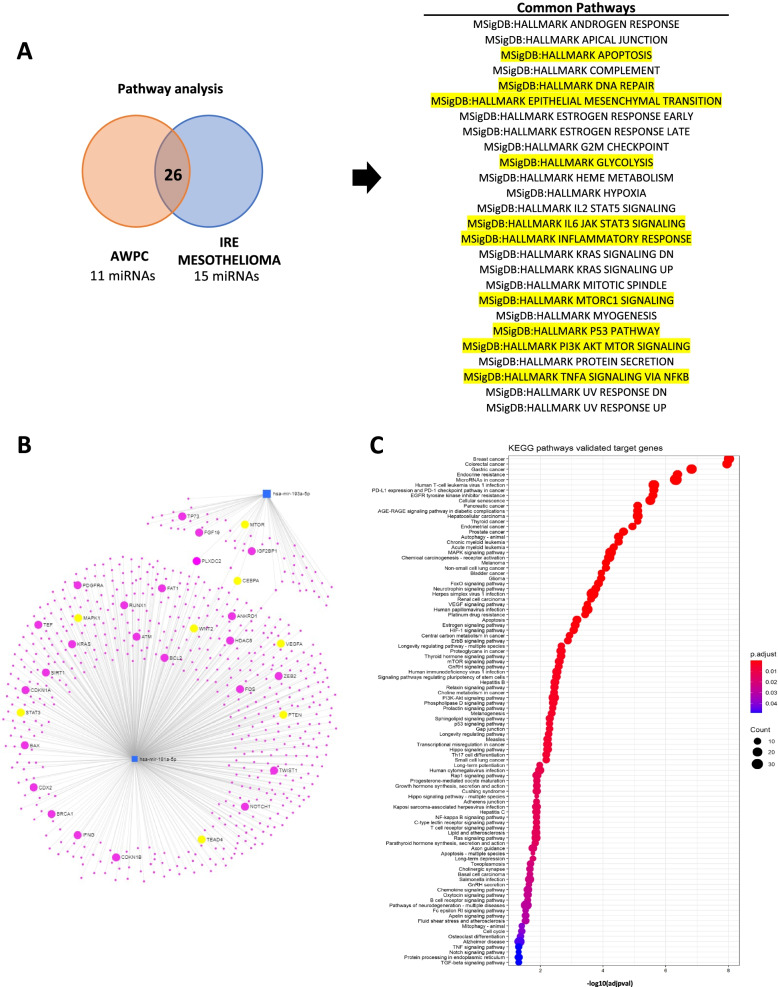
**A** (left side) Workflow of the pathway analysis performed by combining the predicted pathways of 11 miRNAs signature with those of 15 top ranking miRNAs of IRE mesothelioma cohort. The 26 common pathways are listed in the table on the right. Highlighted those pathways with a reported involvement in mesothelioma occurrence. **B** Graphical view of a network built by the validated targets of miR-181a-5p and miR-193a-5p. **C** Bubble plot containing specific ontological groups of miR-193a-5p and miR-181a-5p validated targets. The size of each bubble reflects the number of validated targets. The colours of the bubbles display the *p*-values. p with correction < 0.05

Then, we proceeded in the analysis by looking at the Molecular Signatures Database (MSigDB) to see which genes and their functional characteristics were targeted by the two different identified miRNAs signature (Table 3). Intriguingly, we found 26 common pathways from which those highlighted are closely related to mesothelioma development (Fig. 2A).

Subsequently, we went back to the differences in miRNA expression between the two groups of observations (those APWC derived miRNAs and those differentially expressed between benign and malignant tissue). In this further phase of the validation study, we added, as a third group of observations, the miRNAs expression reported by the TCGA mesothelioma cohort.

In the complex comparison of these three different datasets, we identified two miRNAs, miR-181a-5p and miR-193a-5p, that resulted to be the only ones recurrently expressed in all three datasets. They were listed among those miRNAs differently expressed after AWPC treatment, they were differently expressed between benign and malignant mesothelial lesions, and they were significantly expressed in the TCGA mesothelioma cohort as well.

At that stage of the investigation, we assessed their validated targets (Fig. 2B) and the related pathways (Fig. 2C). The in-silico analysis revealed that several targets triggered by miRNA-181a-5p and 193a-5p were involved in mesothelioma development. In particular, we found proteins involved in the regulation of pathways aberrantly activated in mesothelioma, such as MAPK, PI3K-Akt and mTOR signalling pathways [4, 11, 12], as well as the EGFR [13], Hippo [14, 15], epithelial to mesenchymal transition [16] and NF-kappa B signalling pathways [17, 18]. While miRNA-181-5p exerts an inhibitor regulation on those pathways, thereby eliciting a tumor-suppressor effect, miRNA 193-5p had an inverse role. To corroborate this evidence, we observed in the TCGA database that miRNA-181-5p is significantly and inversely related to mesothelioma stages of severity (higher expression for lower stages), while miRNA 193-5p had again the opposite, although not significant, relation (Supplementary Fig. 1B and C).

Collectively, these findings document that APWC-serum modulated miRNAs might have important implications in the transition from asbestosis to mesothelioma development.

### Compliance and adverse reactions

Among the 18 analyzable subjects, the compliance with study drug was excellent (Table 4). Of the 4 who withdrew, one never took any study drug. Of the 21 patients who ingested study drug, 11 experienced at least one AR (Table 5). None were considered serious ARs. However, the study drug was suspected as the cause in 3 of these subjects, and all 3 withdrew from the study (2 with diarrhea, one with mild light-headedness). There was no study drug related ARs in the 18 patients who completed the trial.

**Table 4:** Compliance with Medications

Characteristic	Completed Trial (*n* = 18)	Withdrew (*n* = 4)
Pills Taken: median, range	351 (323–375)	0, < 16, 36, 172
Compliance: median, range	98% (90–104)%	0%, < 5%, 10%, 48%

**Table 5:** List of Adverse Reactions

*Adverse Reactions in Analyzable Group (n* = *18*)											
**ID**	**Reg Date**	**Day**	**AR Description**	**AR#**	**Start** **Date**	**Stop** **Date**	**On-** **going**	**Grade**	**Action**	**Outcome**	**Relation**
1	26-Nov-14	85	Fell—skinned knee	1	19-Feb-15	19-Feb-15		mild	no change	resolved	**unrelated**
1		88	Fell—sore hip	2	22-Feb-15	22-Feb-15		mild	no change	resolved	**unrelated**
2	26-Nov-14	24	Infection—respiratory	1	20-Dec-14	17-Jan-15		moderate	no change	resolved	**unrelated**
2		28	**Diarrhea**	2	24-Dec-14	27-Dec-14		mild	no change	resolved	**unrelated**
2		68	Decreased appetite	3	02-Feb-15		**Yes**	moderate	no change	chronic	**doubtful**
3	06-Jan-15	40	Infection—respiratory	1	15-Feb-15	01-Mar-15		moderate	no change	resolved	**unrelated**
8	09-Feb-15	86	Insect bite	1	06-May-15		**Yes**	mild	no change	chronic	**unrelated**
10	09-Mar-15	50	Phlegm	1	28-Apr-15		**Yes**	mild	no change	chronic	**unrelated**
14	27-Mar-15	61	Hand Injury	1	27-May-15	23-Jun-15		moderate	no change	resolved	**unrelated**
14		61	**Diarrhea**	2	27-May-15	01-Jul-15		mild	no change	chronic	**unrelated**
16	13-Apr-15	16	Hypertension—unstable	1	29-Apr-15		**Yes**	moderate	no change	chronic	**unrelated**
21	20-Jul-15	7	Constipation	1	27-Jul-15	03-Aug-15		mild	no change	resolved	**unrelated**
*Adverse Reactions in 3 Patients Who Took Study Drug and Then Withdrew*											
**ID**	**RegDate**	**Day**	**AR Description**	**AR#**	**Start** **Date**	**Stop** **Date**	**On-** **going**	**Grade**	**Action**	**Outcome**	**Relation**
5	08-Jan-15	0	Lightheaded	1	08-Jan-15	**13-Jan-15**		mild	**Perm Disc**	resolved	**probable**
19	01-Jun-15	3	**Diarrhea**	1	04-Jun-15	**22-Jun-15**		mild	**Perm Disc**	resolved	**probable**
20	02-Jul-15	30	**Diarrhea**	1	01-Aug-15	09-Aug-15		**severe**	no change	resolved	**possible**
20		30	Pain—Back	2	01-Aug-15		**Yes**	moderate	no change	chronic	**unrelated**
20		50	**Diarrhea**	3	21-Aug-15	**30-Aug-15**		**severe**	**Perm Disc**	resolved	**probable**

## Discussion

In the present study we tested the effect of an artichoke phytocomplex on both mesothelin serum level and circulating miRNA expression in patients diagnosed with lung asbestosis and benign asbestos-related pleural disease. The study included 18 patients who were recruited over a nine-month-period and treated for three months.

The clinical model used for the present study represented a proxy model to study AWPC efficacy in mesothelioma prevention and treatment. As mentioned, the advanced disease stage of mesothelioma in *in-patients* at the St Joseph Hospital, where the trial was conducted, did not allow the study implementation on actual mesothelioma cases. Thus, we used as disease model, lung asbestosis and benign mesothelium plaques instead of mesothelioma as pathological condition associated with exposure to asbestos. In terms of outcome measures, in absence of stronger biomarkers of mesothelioma therapeutic efficacy, we decided to consider serum mesothelin levels (primary outcome) and the miRNA expression profile (secondary outcome).

Mesothelin is a cell-adhesion glycoprotein that is over-expressed in MPM [19]. Serum mesothelin levels are elevated in patients with MPM in comparison to asbestos-exposed controls [20]. A meta-analysis reported a sensitivity of 32% for serum mesothelin with 95% specificity [21] which makes it a weak biomarker for mesothelioma detection and a problematic biomarker of efficacy. The study compliance, as reported, was excellent and the phytocomplex dosage was based on the experimental studies [4] translated, for human use, by the cited USA-FDA tables. In that experimental study we observed that AWPC treatment strongly reduced cell growth, migration, and tumor engraftment of mesothelioma in both *in-vitro* and *in-vivo* experiments. Thus, we believe that mesothelin as a poor-performing biomarker could, at least in part, be responsible for the weakness of the main outcome results. Additional limitations of our study that warrant consideration are the clinical model that does not directly address the exact AWPC effect on mesothelioma lesions and the limited sample size.

When we looked at the miRNAs as secondary aim, the results seemed to be more interesting. On the overall 2571 human miRNAs per subject, unsupervised informatic analysis allowed us to identify, across all study participants, 11 miRNAs significantly dysregulated between the baseline and the AWPC treatment. It was interesting to note that the targeted functional characteristics of the identified 11 miRNAs were connected to cancer development, metabolism and inflammation: all are important pathways activated in mesothelioma development and progression. From the validation study we conducted on these data, we were able to deeply characterize the functional connections between miRNAs, in particular two miRNAs, miR-181a-5p and miR-193a-5p, target of AWPC and modulators of mesothelioma development.

MiRNAs have roles as tumor suppressors and oncogenes and they are modulated by a number of agents and life-style factors. Therefore, these molecules could be considered as novel biomarkers of cancer risk and responsive therapeutic targets for more effective management of human cancers. Thus, our study results may indicate that AWPC could have an actual impact on relevant pathways connected to mesothelioma etiology and development.

In the study we did not observe any severe adverse effects with only reported mild effects mainly related to GI symptoms. This lack of severe side effects of AWPC and the evidence of its impact on relevant developmental pathways of mesothelioma lead us again to consider this phytocomplex as a potential safeguard with preventive or therapeutic potential.

## Conclusions

There has been very limited research on mesothelioma prevention and early treatment. The most important result of our study is the indication that it would be socially and scientifically relevant investigating further the role of chemoprevention in preventing the occurrence of mesothelioma. New and much larger clinical studies based on follow-up of workers exposed to asbestos and on the early detection of malignant lesions are needed. The integration of a correct prospective design, the implementation of an integrated combination of individual risk molecular biomarkers and druggable targets of early neoplastic on-set together with the identification of innovative anti-cancer agents will allow new chemoprevention strategies to control and potentially defeat mesothelioma development.

## Supplementary Information

**Additional file 1: Supplementary Figure 1.** **A.** (left side) Graphical view of a network built by the validated targets of the 11 differently expressed miRNAs signature. Targets of each miRNA are graphed with a specific colour. Orange and purple colours indicate targets triggered by three or two miRNAs respectively. (right side) Graphical view of miR-193a-5p (blue) targets, miR-181a-5p (yellow) and their common targets (purple). **B-C**. Boxplot analysis representing miR-181a-5p (B) or miR-193a-5p (C) log2 expression levels among the different mesothelioma stages from the mesothelioma TCGA dataset.

**Additional file 2: Supplementary Table 1.** List of 11 significantly dysregulated miRNAs between the baseline and after the three months of AWPC treatment.

**Additional file 3: Supplementary Table 2.** List of 15 significantly dysregulated miRNAs between unmatched tissues from benign pleural lesions (same type we used as an inclusion criterion in the trial) derived from 12 subjects and mesothelioma-derived tissues collected from 29 patients.

## Data Availability

All data generated or analysed during this study are included in this manuscript and its supplementary information files.

## Abbreviations

AWPC: Artichoke freeze-dried extract
MPM: Malignant Pleural mesothelioma
CBC: Complete blood count
ALT: Alanine aminotransferase
AST: Aminotransferase
TCGA: The Cancer Genome Atlas
MAPK: Mitogen-activated protein kinase
PI3K: PhosphatidylInositol 3-Kinase
Akt: Protein kinase B
mTOR: Mammalian target of rapamycin
EGFR: Epidermal growth factor receptor
AR: Adverse Reactions
GI: Gastrointestinal
