# Supplementary figures and images for: Artichoke phytocomplex modulates serum microRNAs in patients exposed to asbestos: a first step of a phase II clinical trial
Source: J Exp Clin Cancer Res. 2022 Aug 20;41:255. doi: 10.1186/s13046-022-02455-6 (PMC9391647; doi:10.1186/s13046-022-02455-6)

Supplementary Figure 1

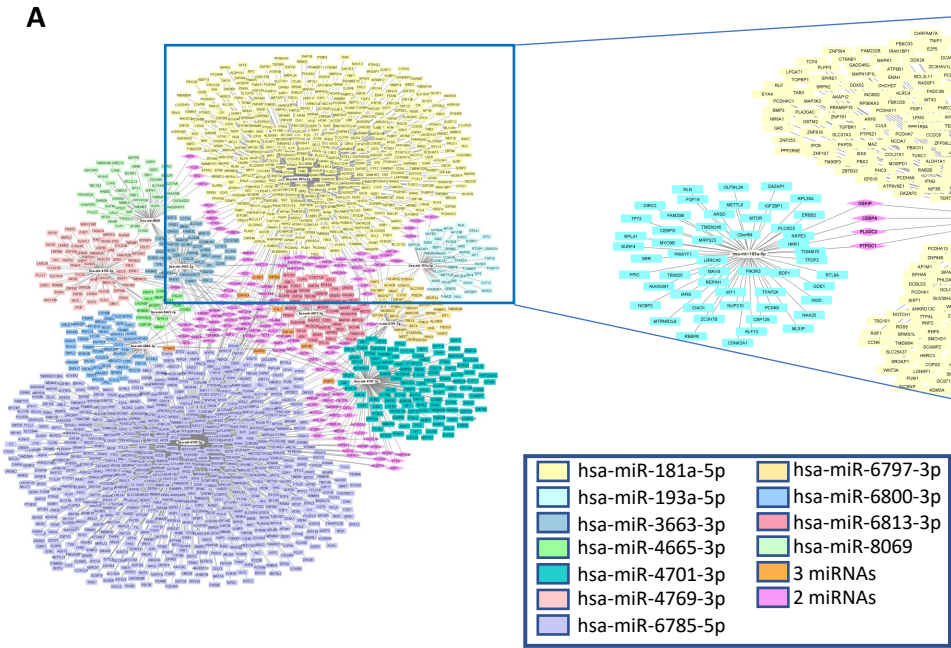

**B**

TCGA MESOTHELIOMA

miR-181a-5p,  $p=0.036855$

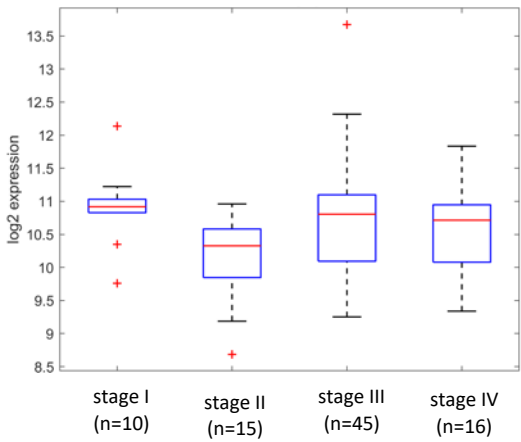

**C**

TCGA MESOTHELIOMA

miR-193a-5p,  $p=0.19268$

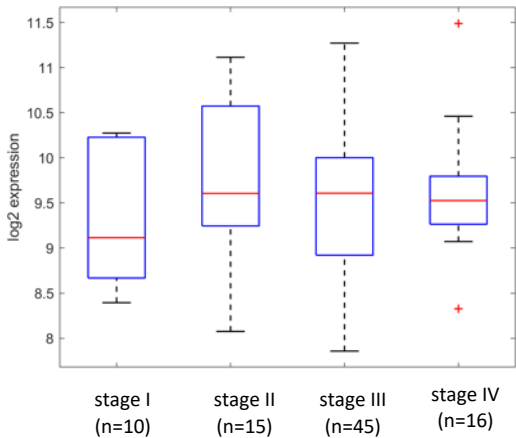

Supplement: Supplementary file 1 — Additional file 1: Supplementary Figure 1. A. (left side) Graphical view of a network built by the validated targets of the 11 differently expressed miRNAs signature. Targets of each miRNA are graphed with a specific colour. Orange and purple colours indicate targets triggered by three or two miRNAs respectively. (right side) Graphical view of miR-193a-5p (blue) targets, miR-181a-5p (yellow) and their common targets (purple). B-C. Boxplot analysis representing miR-181a-5p (B) or miR-193a-5p (C) log2 expression levels among the different mesothelioma stages from the mesothelioma TCGA dataset. [file 13046_2022_2455_MOESM1_ESM.pdf]
